# Supplementary material for: Non-invasive intradermal imaging of cystine crystals in cystinosis
Source: PLoS One. 2021 Mar 4;16(3):e0247846. doi: 10.1371/journal.pone.0247846 (PMC7932553; doi:10.1371/journal.pone.0247846)
Supplement: S1 File — (DOCX) [file pone.0247846.s002.docx]

**Supplementing Information**

**Supplementary Methods**

Image analysis: Automated segmentation, selection and measurement of crystal-like structure area**.** Using ImagePro Premier, image stacks were initially calibrated (1 μm = 1.25 pixel), before first highlighting the total crystals by selecting all bright objects that passed the following filters: area between 9-600 μm^2^, roundness between 0-3, minimum hole area ratio of 0.95 and minimum perimeter ratio of 0.9 (S1 Fig., Step 1). To facilitate selecting larger contiguous areas of skin structure representing the dermal papillae, images were then adjusted to a final setting of 55 contrast and 0.8 gamma, followed by a low-pass filter (3 passes, strength 50) and finally a close filter (5 passes). Regions of skin structure at least 200 μm^2^ were then selected from the adjusted image (Step 2). Total crystal and skin structure regions of interest (ROIs) were both masked (Step 3). In order to enable selection of crystals alone, the intensity of the crystal mask was reduced by ⅔, while the skin structure mask was reduced by ½. Adjusted masks were then combined, and the dimmest elements corresponding to only crystals and not skin structure were selected (Step 4). Finally, we measured the sum area of isolated crystals in each image slice and then normalized this value to the area of the total region excluding the skin structure (Step 5). We report this normalized value adjusted to a useful scale for every slice of the image stack.

Image analysis: 3D reconstruction of the papillary dermis and measurement of crystal-like volumes. Binary composites were generated based on the regions of isolated crystals and skin structures selected in 2D (S1 Fig., step 6). 3D reconstructions were then formed from slice 15-50, a region of roughly 100 μm corresponding to the papillary dermis where maximal crystal accumulation was observed in patients (Step 7). Volumetric crystal measurements were obtained by creating isosurface renderings of isolated crystals and then normalizing the sum volume against the total region, excluding the volume of the skin structure.


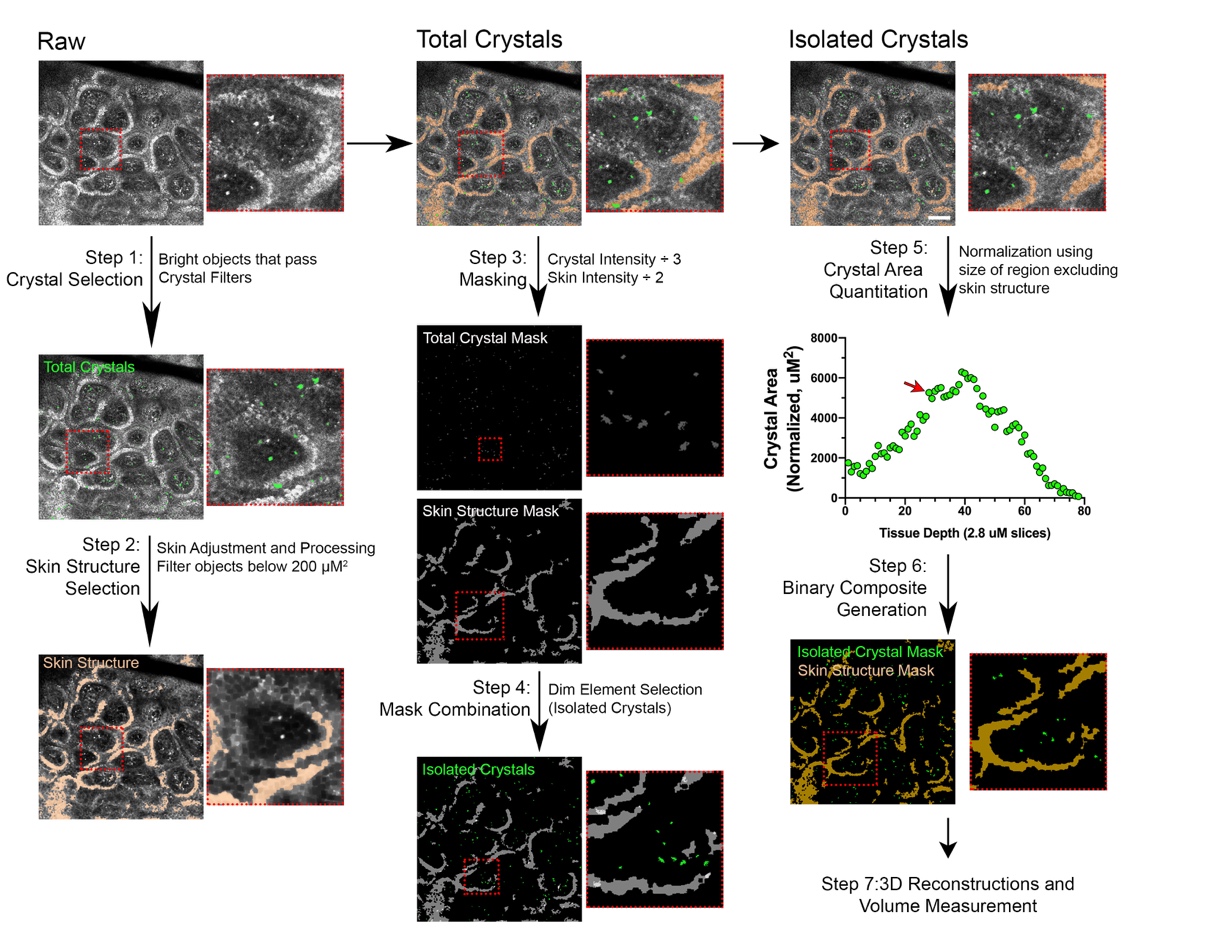


**S1 Fig. Detailed image analysis methodology to quantify intradermal cystine crystal area and volume.** Full step-by-step descriptions including relevant filtration settings may be found in Supplementary Methods.

**S1 Table. Numbers of patients and controls enrolled and analyzed.**

| **Patient Grouping** | **N** | **Control Grouping** | **N** |
| --- | --- | --- | --- |
| Total Enrolled | 70 | Total Enrolled | 31 |
| Patients Genotyped | 51 |  |  |
| Total Imaged | 57 | Total Imaged | 27 |
| Total Stacks Acquired | 83 | Total Stacks Acquired | 38 |
| Imaged in 2018 | 39 | Imaged in 2018 | 17 |
| Imaged in 2019 | 44 | Imaged in 2019 | 21 |
| Medical Records Acquired | 47 |  |  |
| Infantile Cystinosis Records | 43 |  |  |


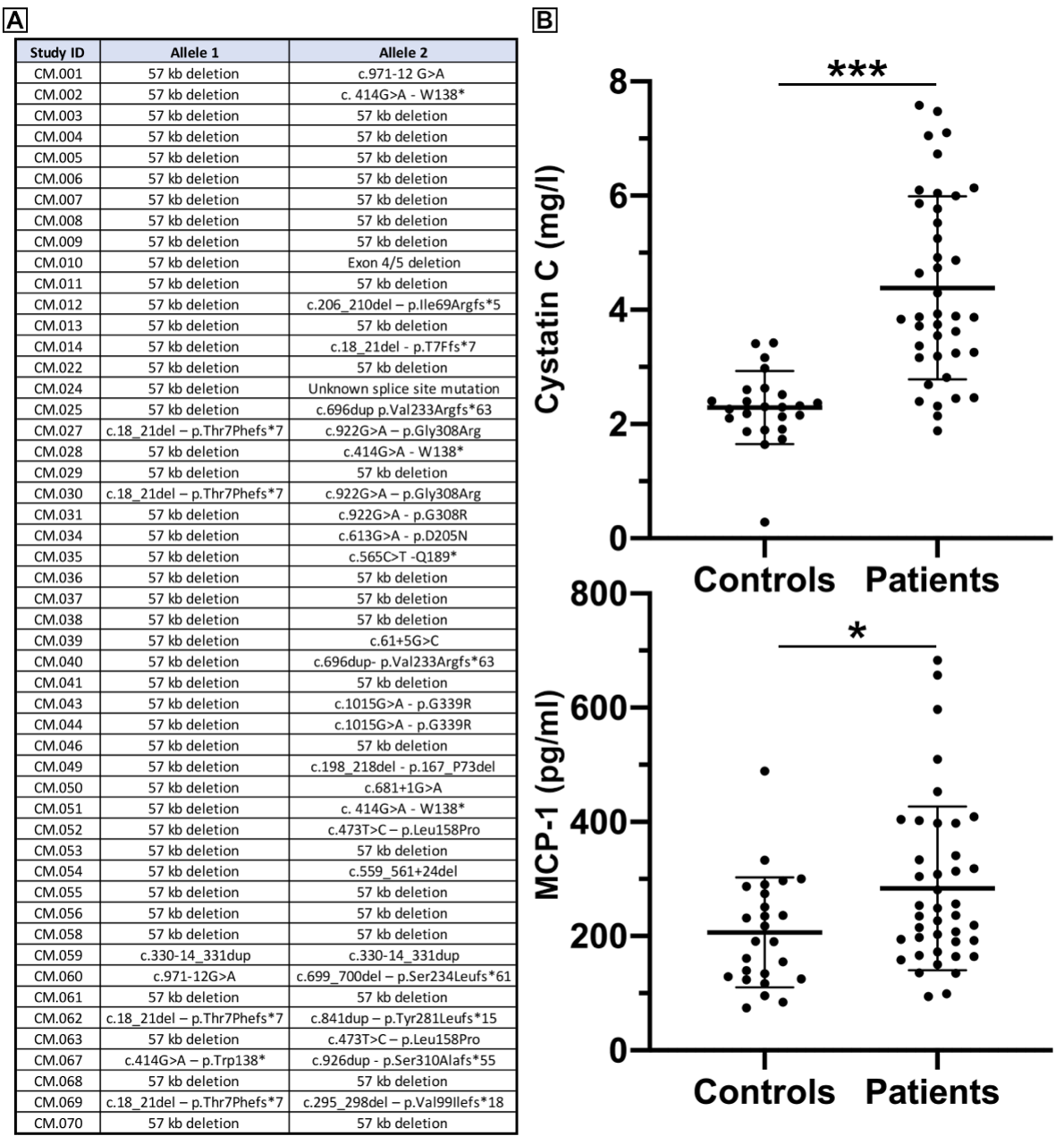


**S2 Fig.** Genetics and ELISA. **A**, *CTNS* mutations were determined using buccal cells from cystinosis patients. PCR and Sanger sequencing were employed to determine *CTNS* mutations listed. **B**, Cystinosis patients have elevated levels of renal function and inflammatory markers in serum. Box-and-whisker plots show serum concentrations of the renal marker Cystatin C (patients (n=40) and controls (n=24)) and inflammatory marker MCP-1 (patients (n=41) and controls (n=25)) +/- SD as determined by ELISA.

**S2 Table. Summary statistics for crystal area accumulation in 2D for cystinotic patients compared to healthy controls.**

**S3 Table. Complete demographic and clinical characteristics of all study patients. ***

| **Characteristic** |  | **Value** | **Units** |
| --- | --- | --- | --- |
| Age (Median [range]) |  | 11 (0.8-54) | years |
| Gender (%) | Female | 22 (51.2%) |  |
|  | Male | 21 (48.8%) |  |
| Type of Cystinosis (%) | Infantile  Juvenile | 43 (0.91%)  4 (0.09%) |  |
| Kidney Transplant (%) | No | 34 (79.1%) |  |
|  | Yes | 9 (20.9%) |  |
| Cysteamine Medication (%) | None | 2 (4.7%) |  |
|  | Cystagon | 8 (18.6%) |  |
|  | Procysbi | 33 (76.7% |  |
| Cysteamine Medication Dosage (Mean[SD]) |  | 1185.32 (694.0) | mg |
| CKD (patients without kidney transplant) (%) | No | 13 (38.2%) |  |
|  | Stage 1 | 4 (11.8%) |  |
|  | Stage 2 | 8 (23.5%) |  |
|  | Stage 3 | 7 (20.6%) |  |
|  | Stage 4 | 2 (5.9%) |  |
| Glomerular Filtration Rate (GFR) (Mean[SD]) |  | 70.99 (37.40) | mL/min |
| Hypothyroidism (patients age>=9) (%) | No | 12 (60%) |  |
|  | Yes | 8 (40%) |  |
| Hyperparathyroidism (%) | No  Yes | 29 (72.5%)  11 (27.5%) |  |
| Thyroid Stimulating Hormone (TSH) (Mean[SD]) |  | 3.7 (6.11) | IU/L |
| Parathyroid Hormone (PTH) (Mean[SD]) |  | 47.06 (43.56) | pg/mL |
| T4 |  | 4.64 (12.97) | ng/dL |
| nCCV (Mean [SD]) |  | 1633.5 (806.5) | μm^3^ |
| Granulocyte cystine level (median[IQR]) |  | 1.07 (0.8-1.5) | nmol/mg |
| Fanconi Syndrome (%) | No | 10 (23.3%) |  |
|  | Yes | 33 (76.7%) |  |
| Polydipsia (%) | No | 27 (64.3%) |  |
|  | Yes | 15 (35.7%) |  |
| Polyuria (%) | No  Yes | 14 (35%)  26 (65%) |  |
| Acidosis (%) | No  Yes | 19 (45.2%)  23 (54.8%) |  |
| Diarrhea (%) | No  Yes | 30 (75%)  10 (25%) |  |
| Vomiting (%) | No  Yes | 18 (45%)  22 (55%) |  |
| Muscle Weakness (%) | No  Yes | 34 (85%)  6 (15%) |  |
| Dysphagia (%) | No  Yes | 33 (82.5%)  7 (17.5%) |  |
| Corneal Photophobia (%) | No  Yes | 25 (62.5%)  15 (37.5%) |  |
| Photosensitivity (%) | No  Yes | 34 (82.9%)  7 (17.1%) |  |
| Corneal Cystine Crystals (%) | No  Yes | 17 (41.5%)  24 (58.5%) |  |
| Rachitism (%) | No  Yes | 24 (60%)  16 (40%) |  |
| Genu Valgus (%) | No  Yes | 30 (75%)  10 (25%) |  |
| Pulse (Mean[SD]) |  | 99 (24.7) | bpm |
| BP Systolic (Mean[SD]) |  | 107.6 (17.97) | mmHg |
| BP Diastolic (Mean[SD]) |  | 62.6 (10.97) | mmHg |
| Pulse Pressure (Mean[SD]) |  | 43.3 (26.97) | mmHg |
| Height (Mean[SD]) |  | 118.3 (36.08) | cm |
| Weight (Mean[SD]) |  | 34.3 (29.57) | kg |
| Body Mass Index (BMI) (Mean[SD]) |  | 17.6 (1.25) | kg/m^2^ |
| Use of Growth Hormone | No  Yes | 28 (68.3%)  13 (31.7%) |  |
| Hemoglobin (Mean[SD]) |  | 17.7 (25.4) | g/dL |
| Hematocrit (Mean[SD]) |  | 37.4 (4.15) | % |
| Platelet Count (Mean[SD]) |  | 306 (11.74) | 10^9^/L |
| White Blood Cell (WBC) Count (Mean[SD]) |  | 8.23 (3.16) | k/μL |
| Red Blood Cell (RBC) Count (Mean[SD]) |  | 4.4 (0.49) | 10^6^/ μL |
| Neutrophil Count (Mean[SD]) |  | 4.07 (1.69) | 10^3^/ μL |
| Lymphocyte Count (Mean[SD]) |  | 3.48 (5.07) | 10^3^/ μL |
| Monocyte Count (Mean[SD]) |  | 0.69 (0.38) | 10^3^/ μL |
| Eosinophil Count (Mean[SD]) |  | 0.28 (0.35) | 10^3^/ μL |
| Basophil Count (Mean[SD]) |  | 0.04 (0.04) | 10^3^/ μL |
| Percent Neutrophil (Mean[SD]) |  | 53.27 (13.14) | % |
| Percent Lymphocyte (Mean[SD]) |  | 34.4 (13.88) | % |
| Percent Monocyte (Mean[SD]) |  | 8.46 (2.34) | % |
| Percent Eosinophil (Mean[SD]) |  | 2.78 (2.03) | % |
| Percent Basophil (Mean[SD]) |  | 0.57 (0.47) | % |
| Blood Urea Nitrogen (BUN) (Mean[SD]) |  | 19.09 (14.47) | mg/dL |
| Serum Creatinine (Mean[SD]) |  | 1.87 (5.12) | mg/dL |
| Serum Cystatin (Mean[SD]) |  | 1.69 (0.51) | mg/L |
| Serum Sodium (Mean[SD]) |  | 139.17 (2.61) | mmol/L |
| Serum Chloride (Mean[SD]) |  | 101.9 (16.87) | nmol/L |
| Serum Potassium (Mean[SD]) |  | 3.88 (0.55) | mmol/L |
| Serum Bicarbonate (Mean[SD]) |  | 22.73 (2.67) | mmol/L |
| Albumin (Mean[SD]) |  | 4.14 (0.41) | g/dL |
| Serum Phosphorus (Mean[SD]) |  | 3.64 (1.5) | mg/dL |
| Alkaline Phosphatase (Mean[SD]) |  | 233.82 (131.65) | U/L |
| Aspartate Aminotransferase (Mean[SD]) |  | 31.86 (19.81) | U/L |
| Serum Calcium (Mean[SD]) |  | 8.21 (2.57) | mg/dL |
| Serum Glucose (Mean[SD]) |  | 83.12 (29.64) | mg/dL |
| Total Protein (Mean[SD]) |  | 6.83 (0.66) | g/L |
| Total Bilirubin (Mean[SD]) |  | 0.53 (0.29) | mg/dL |
| Anion Gap (Mean[SD]) |  | 11.88 (3.6) | mEq/L |
| Iron (Mean[SD]) |  | 88.97 (39.37) | μg/dL |
| Vitamin D (Mean[SD]) |  | 43.74 (17.62) | mg/mL |
| Specific Gravity (Urine) (Mean[SD]) |  | 0.98 (0.18) |  |
| Urine pH (Mean[SD]) |  | 7.16 (1.24) |  |
| Urine Protein (%) | Negative  Positive | 2 (6.7%)  28 (93.3%) |  |
| Urine Glucose (%) | Negative  Positive | 13 (40.6%)  19 (59.4%) |  |
| Urine Ketones (%) | Negative  Positive | 17 (54.8%)  14 (45.2%) |  |
| Urine Leukocyte Esterase (%) | Negative  Positive | 28 (90.3%)  3 (9.7%) |  |
| Urine Blood (%) | Negative  Positive | 14 (48.3%)  15 (51.7%) |  |
| Urine Creatinine (Mean[SD]) |  | 23.93 (27.27) | mg/dL |
| *: No filters or exclusions have been applied to this table. |  |  |  |


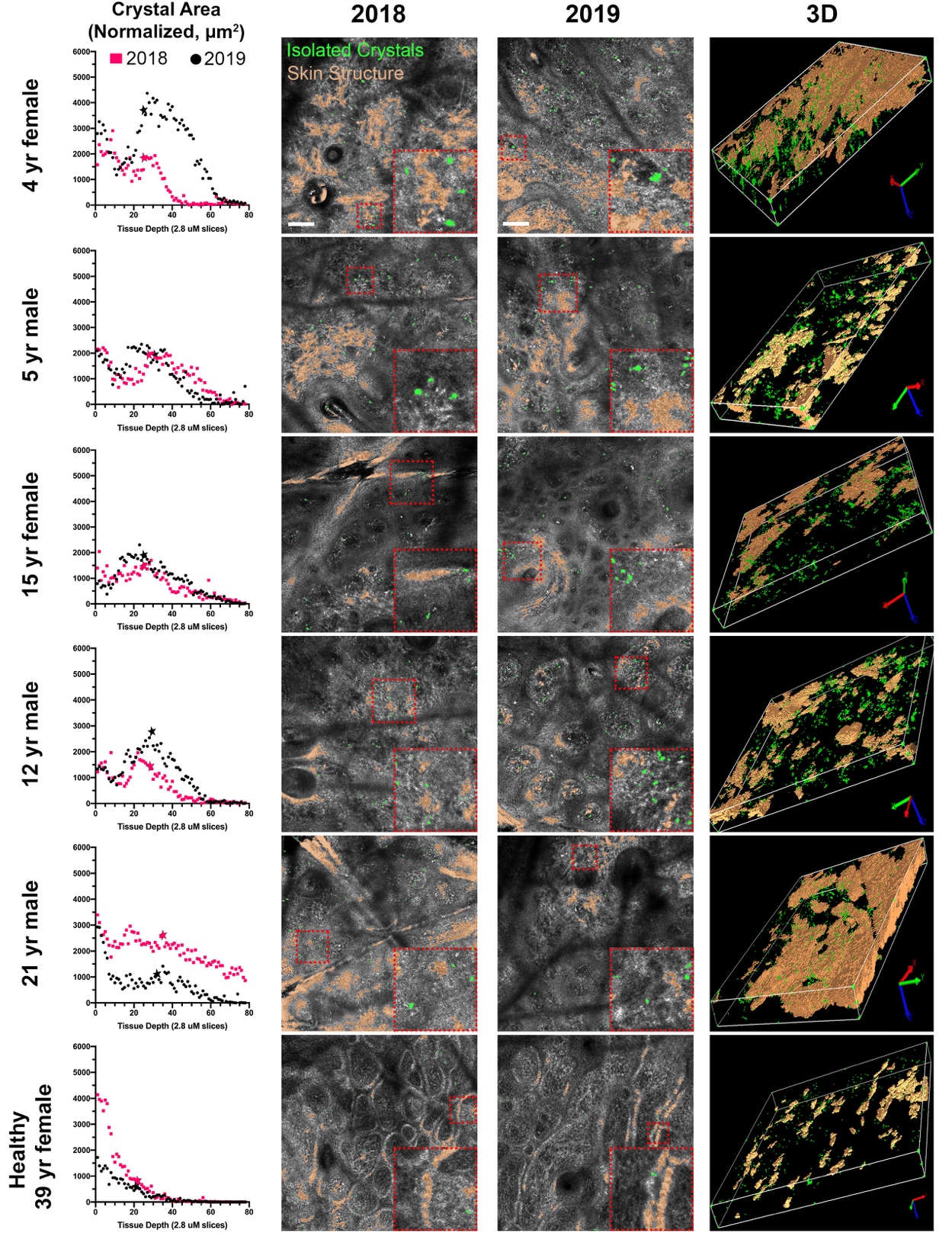


**S3 Fig.** **Additional examples of longitudinal intradermal imaging of cystinotic children, adolescents and adults compared to healthy control subject.**  XY scatterplot at left depicts sum crystal area at each slice from two years of quantification. Stars represent which slice is depicted as a representative example from each year to right. Scale bar = 100 μm. Far right depicts 3D reconstruction of the papillary dermis region from most recent image acquisition.


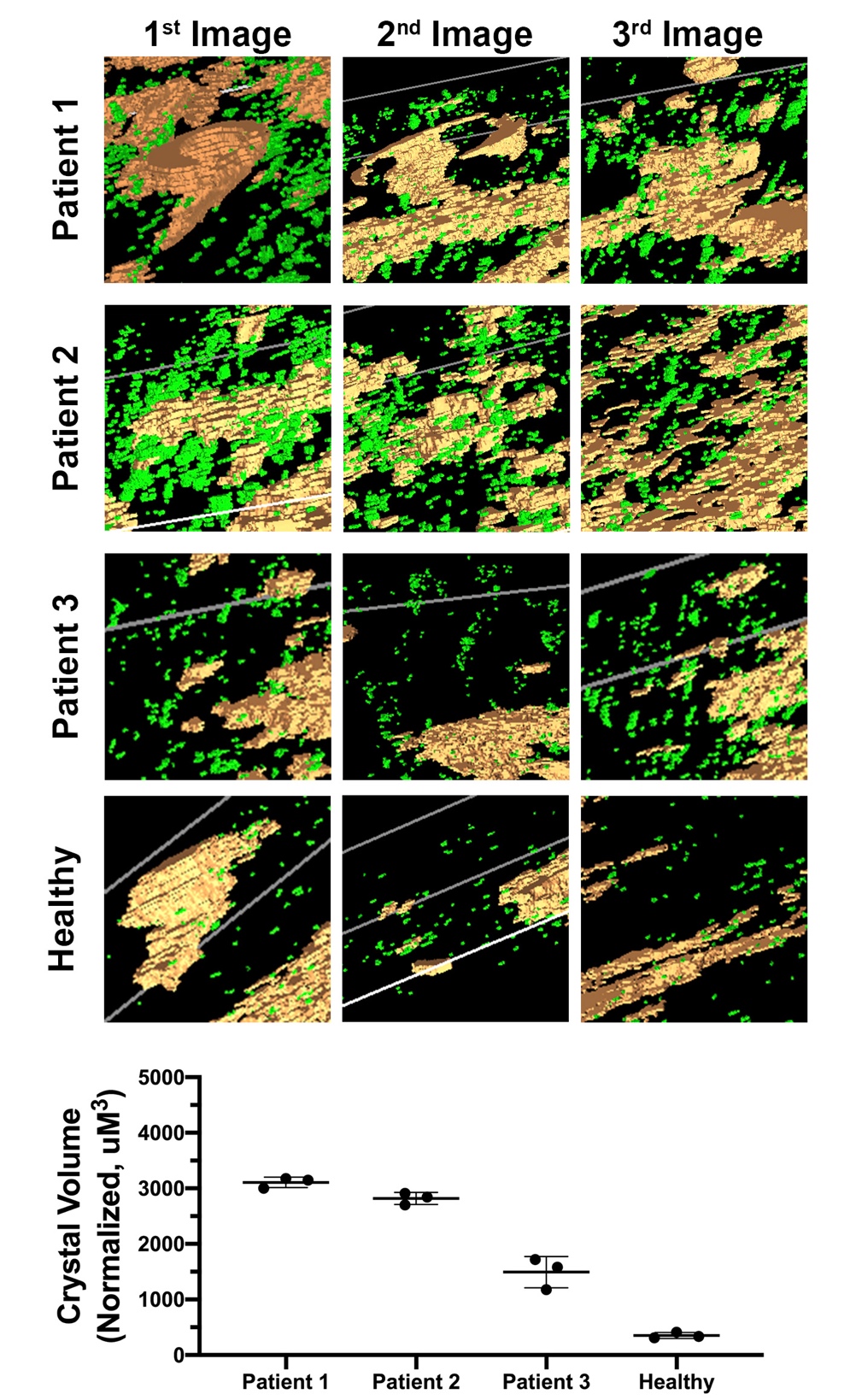


**S4 Fig.** Intradermal imaging and volumetric crystal analysis is reproducible. Three image stacks were acquired at the same time and location sequentially from cystinosis patients and healthy controls. Representative regions of 3D reconstructions are shown. Crystals displayed in green while skin structure is shown in orange. Box-and-whisker plot displays normalized sum crystal volume for each subject triplicate.
