# Supplementary material for: Non-invasive intradermal imaging of cystine crystals in cystinosis
Source: PLoS One. 2021 Mar 4;16(3):e0247846. doi: 10.1371/journal.pone.0247846 (PMC7932553; doi:10.1371/journal.pone.0247846)
Supplement: Attachment — Submitted filename: Plos One.pdf [file pone.0247846.s011.pdf]

This study highlights promising new technology to non-invasively follow disease status in cystinosis. There are no previous studies done with this technology for cystinosis patient. Manuscript is technically sound and data support the conclusion but below are some comments:

1. Include final sample sizes used in statistics section within methods. For example it was not clear what sample size was used for ELISA? Mention if replicates were performed?
2. Point out in H&E staining image perivascular chronic inflammatory cells in figure 1A as mentioned in page 10.
3. Figure 2 C states that “Preliminary slices were excluded due to signal background these skin layers in healthy controls.” This statement is not clear, clarify this statement further, What is the tissue depth of these excluded slices? Were they present in the starred region? What is justification to remove them? Will this effect results if we include them, if yes is there background in patients’ images as well at this location? By excluding this are we still comparing pateints vs controls in similar experimental setup/ conditions?
4. Indicate sample size used for figure 3D
5. Typographical error in page 14 second paragraph line 3. Correct hrough to “Through”
6. Conclusion is based on results observed but can this methodology be applied to people with with dark skin people? Are there any other challenges with this methodology?
